# Effect of breeding on nitrogen use efficiency-associated traits in oilseed rape

**DOI:** 10.1093/jxb/erz044

**Published:** 2019-02-09

**Authors:** Andreas Stahl, Paul Vollrath, Birgit Samans, Matthias Frisch, Benjamin Wittkop, Rod J Snowdon

**Affiliations:** 1 Department of Plant Breeding, IFZ Research Centre for Biosystems, Land Use and Nutrition, Justus Liebig University, Giessen, Germany; 2 Department of Biometry and Population Genetics, IFZ Research Centre for Biosystems, Land Use and Nutrition, Justus Liebig University, Giessen, Germany

**Keywords:** *Brassica napus*, breeding, hybrid varieties, nitrogen uptake, nitrogen utilization, selection, thousand seed weight, yield

## Abstract

Oilseed rape is one of the most important dicotyledonous field crops in the world, where it plays a key role in productive cereal crop rotations. However, its production requires high nitrogen fertilization and its nitrogen footprint exceeds that of most other globally important crops. Hence, increased nitrogen use efficiency (NUE) in this crop is of high priority for sustainable agriculture. We report a comprehensive study of macrophysiological characteristics associated with breeding progress, conducted under contrasting nitrogen fertilization levels in a large panel of elite oilseed rape varieties representing breeding progress over the past 20 years. The results indicate that increased plant biomass at flowering, along with increases in primary yield components, have increased NUE in modern varieties. Nitrogen uptake efficiency has improved through breeding, particularly at high nitrogen. Despite low heritability, the number of seeds per silique is associated positively with increased yield in modern varieties. Seed weight remains unaffected by breeding progress; however, recent selection for high seed oil content and for high seed yields appears to have promoted a negative correlation (*r*= –0.39 at high and *r*= –0.49 at low nitrogen) between seed weight and seed oil concentration. Overall, our results reveal valuable breeding targets to improve NUE in oilseed rape.

## Teaser

Enhanced nitrogen uptake and number of seeds per plant are associated with breeding-driven improvement of nitrogen use efficiency. Direct or indirect selection decisions led to a negative correlation between seed weight and oil content.

## Introduction

Agricultural production must increase global crop yields dramatically in order to match growing demand from a predicted world population of ~9 billion people by 2050 (Tilman *et al.*, 2002). Since arable land is limited, yield improvements per unit area are of utmost importance. In most regions, this is associated with fertilization with nitrogen (N), the quantitatively most important nutrient required for crop growth. However, negative environmental impacts of N fertilization, such as pollution of waterways through nitrate leaching and runoff (Barton and Colmer, 2006), greenhouse gas emissions (Walter *et al.*, 2015), and potential reduction of biodiversity (Suding *et al.*, 2005; Clark and Tilman, 2008), necessitate adjustments in agricultural farming practice to reduce environmental damage (Rockström *et al.*, 2009; Steffen *et al.*, 2015). Solving the conflict between the need for yield increases, without compromising crop product quality or ecosystem health through excessive N losses, necessitates tremendous enhancement of nitrogen use efficiency (NUE) in major crops.

Oilseed rape (*Brassica napus* L.) is the most heavily produced oil crop in northern Europe, Canada, China, and Australia. All major ecogeographical forms of oilseed rape (winter, spring, and semi-winter types) require high N fertilization at the beginning of vegetation, but the crop frequently suffers from an N balance surplus at maturity (Aufhammer *et al.*, 1994; Rathke *et al.*, 2006; Sieling and Kage, 2006; Bouchet *et al.*, 2016). Recent studies have revealed considerable scope for improvement in NUE of oilseed rape (Weiser *et al.*, 2017). This can be partially achieved by approaches that simultaneously consider crop management (Hegewald *et al.*, 2016, 2017; Kirkegaard *et al.*, 2016), more accurate prediction of N requirement (Barlog and Grzebisz, 2004*a*, b; Henke *et al.*, 2007), timing of N fertilization (Berry and Spink, 2009; Gombert *et al.*, 2010), and more contemporary, precise application of N fertilizer. On the other hand, appropriate use of genetic variation to increase yield has been recognized as a major driver of sustainable production. In particular, hybrid varieties are thought to be beneficial for an efficient use of N (Gehringer *et al.*, 2007; Kessel *et al.*, 2012; Wang *et al.*, 2016; Stahl *et al.*, 2017). To date, however, detailed data on the causative physiological parameters explaining the superior performance of modern hybrid varieties remain scarce; however, such information is of tremendous relevance for decisions in future breeding.

In general, NUE can be split into two major trait complexes, nitrogen uptake efficiency (NupE) and nitrogen utilization efficiency (NutE; Moll *et al.*, 1982; Good *et al.*, 2004). Earlier studies revealed that genotypic variation in NUE in winter oilseed rape is associated with NupE particularly during generative developmental stages (Berry *et al.* 2010). Furthermore, efficient genotypes were found to show a stay-green phenotype, prolonging the duration of photosynthesis and thus supporting ongoing N uptake even during the generative developmental phase (Schulte auf’m Erley *et al.*, 2007, 2011). Furthermore, a link was discovered between genetic differences in root growth characteristics and N uptake beyond the start of flowering (Kamh *et al.*, 2005; Ulas *et al.*, 2012). Subsequent grafting experiments revealed that the underlying signals were derived from leaves rather than roots (Koeslin-Findeklee *et al.*, 2015).

During seed maturation, N translocation to generative organs becomes a crucial factor (Masclaux-Daubresse *et al.*, 2008). In comparison with wheat or maize, for example, N remobilization is considerably more relevant in oilseed rape (Malagoli *et al.*, 2005; Gombert *et al.*, 2010), where most of the N stored in the seeds originates from remobilized N (Hirel *et al.* 2007). However, in regard to N remobilization, oilseed rape is limited by an asynchrony of N availability of source tissues and demand of sink tissues (reviewed in Bouchet *et al.*, 2016). Thus, remobilization is an important aspect of enhanced NUE in oilseed rape (Ulas *et al.* 2013; Koeslin-Findeklee and Horst, 2016). Berry *et al.* (2010) and Kessel *et al.* (2012) found that under high N conditions, variation in NUE is mainly caused by variation in NutE, while, at low N, differences in NupE gains importance. Koeslin-Findeklee *et al.* (2014) suggest that the superior performance of hybrids is associated with both better NupE and NutE, but not late senescence.

For seed yield—and consequently NUE—plant architecture and yield components (YCs) are further important aspects to consider (Diepenbrock, 2000). Combinations of YCs with different genetic determinants may lead to high-yielding varieties (Cai *et al.* 2016). Indeed, older investigations found that the number of siliques per plant responded most strongly to N supply and was causative for higher yields, whereas neither the number of seeds per silique nor the thousand seed weight (TSW) showed a similar response (Hocking *et al.*, 1997). Asare and Scarisbrick (1995) also related elevated seed yield to a higher number of siliques on the terminal raceme and a higher TSW, but not to the number of seeds per silique. It is known that a higher plant density leads to a lower seed yield per plant but higher TSW (Leach *et al.* 1999), whereas more recently it was demonstrated that plasticity of TSW can compensate deficiencies in other YCs (Labra *et al.*, 2017). However, to our knowledge, no study to date has investigated the extent to which YCs differ between hybrid varieties and open pollinated (OP) varieties.

Here we investigate macrophysiological traits in relation to breeding progress within the last two decades in a panel of 30 elite varieties which had major commercial relevance in Europe between 1989 and 2014, in a comprehensive analysis spanning three locations, two experimental years, three replicates, and two divergent N fertilization levels. We pay particular attention to inadvertent selection of relevant growth parameters as a result of yield selection. The findings help identify potential key traits that should be addressed in breeding programs to boost further NUE improvement in N-demanding crops.

## Materials and methods

### Genetic material

This study comprised 30 European winter oilseed rape varieties, registered between 1989 and 2014, including 20 hybrid varieties and 10 OP varieties. All of the tested varieties possess double-low (00) seed quality (low erucic acid, low glucosinolate) and were among the most widely grown varieties in their period of registration. Varieties were classified as old or modern according to the year of release (see Supplementary Table S1 at *JXB* online).

### Plant cultivation

Field experiments were conducted as described in Stahl *et al.* (2017) at three locations in Germany (Asendorf, ASE; Rauischholzhausen, RHH; and Moosburg, MOS) over two cultivation years (2014–2015 and 2015–2016 cropping seasons). Soil conditions, N availability at the beginning of the fertilization experiments, and net plot sizes are given in Supplementary Table S2. Sowing density was 50 seeds m^–2^ for all varieties at all locations, adjusted for individual germination rates of the different varieties. Experiments consisted of two contrasting N fertilization levels (120 kg N ha^–1^ versus 220 kg N ha^–1^). In the first experimental year in both N treatments 120 kg N ha^–1^ (including N_min_ measured at the end of winter) were applied in the first fertilizer application. Only in the high N treatment was an additional 100 kg N ha^–1^ applied ~1 month later. In the second experimental year, the low N treatment received 65 kg N ha^–1^ with the first fertilizer application, while the high N treatment received 120 kg N ha^–1^. The second N fertilizer application consisted of 55 kg N ha^–1^ in the low N and 100 kg N ha^–1^ in the high N treatment. N fertilizer was not applied before winter in any of the experiments. Experiments were laid out a split plot design for the N level. Within each N level, plots were arranged as an α-lattice with four replicates and eight sub-blocks each. Sulfur (S) fertilization was consistent across both N fertilization levels, either by using ammonium sulfate saltpeter in the same amount across both N levels or by using a separate S-containing fertilizer. The amount of N in ammonium sulfate saltpeter was included in the total N fertilizer budget. Later, N differentiation between N treatments was done by S-free fertilizer. The first three replicates were used for yield and seed quality determination as well as phenotyping for flowering time and plant length. The fourth replicate was used for sampling of plants from the center of the plot for determination of YCs, thus allowing destructive sampling without influencing the yield results from the first three replicates. Oilseed rape development is characterized by an early N uptake which reaches its peak at flowering. Thereafter, due to the switch from the vegetative to the generative growth stages, oilseed rape loses N by insufficient reallocation of N from senescing leaves. Therefore, in both years at field sites MOS and RHH, the entire experiment, including both N levels and replications, was mirrored in two identical copies each (with independent randomization). This allowed a destructive harvest of total plant biomass at the flowering peak of the main racemes (MRs; BBCH 67–69) to enable determination of Nup at both locations. Traits associated with sample collection at flowering at those two locations were classified as category I traits, whereas traits determined at all three locations were classified as category II traits (Supplementary Table S3).

### Determination of nitrogen uptake at flowering (category I traits)

Category I traits were measured at the flowering peak of MRs (BBCH 67–69). From three replicated plots per genotype, five (six for RHH2015) adjacent plants were collected from the same row in the middle of a plot to exclude effects from neighboring plots. Each individual plant was separated into (i) leaves, (ii) stems, and (iii) flowers (including the first developing siliques). Tissues were dried between 70 °C and 80 °C for 72 h and ground to a fine powder after determination of the dry mass. After collection of the five single plants, the entire plant biomass from the core of each plot (RHH, 10.5 m^2^; MOS, 10.2 m^2^) was harvested with a plot forage harvester to determine the entire plant fresh mass. An aliquot of this biomass was used for dry matter concentration determination immediately after harvest. Plant dry mass was calculated by multiplying plant fresh mass by dry matter concentration. All tissues samples were analyzed in triplicate for their N and C concentration according to the Dumas combustion method (Dumas, 1826; Buckee, 1994) using a Vario cube EL elementar analyzer (Elementar Analysesysteme GmbH, Hanau, Germany). In order to calculate the N concentration of individual plant partitions, N and C concentrations were multiplied by the corresponding dry mass, summarized at the whole-plant level. Nup was calculated by multiplying the amount of N at the individual aboveground plant level by the ratio of dry mass per hectare over the individual plant dry mass. Root N was neglected for Nup in this study.

### Determination of category II traits

Flowering time was manually determined as day of the year (doy). Plant length was measured on fully developed plants after the end of flowering in both N treatments. Towards the end of seed filling (~2 weeks before threshing), plants were collected at locations ASE, MOS, and RHH for determination of primary YCs. Three adjacent plants from a middle row of one plot per N treatment were collected and separated into MR and side branches (SBs). On each of these plants (*n*>1000), the numbers of siliques per MR and SBs, respectively, were counted manually (cumulative counts for all SBs). Yield of the MR and the cumulative yield of SBs were measured by weighing dry samples from each plant individually. Thousand seed weight (TSW) was determined based on counting and weighing 100 seeds twice from a representative 100 g aliquot from each of three replicated plots at both N fertilization levels at each location. TSW, seed yield of individual plants, and number of siliques were used to calculate the average number of seeds per silique on the MR and the SBs, separately. Determination of seed yield and seed quality traits was performed as described previously (Stahl *et al.*, 2017). NutE was determined by dividing the seed yield by the Nup. NUE was defined as the seed yield per unit of fertilized N.

### Data analysis

Adjusted means of category I traits were calculated in a two-step approach. First, adjusted means of phenotypic data were calculated for individual N fertilization level and environment, separately, according to the general mixed linear model described in Equation 1.

$${\text{P}}_{iln}=\;\mu \;+g_{i}+{\text{R}}_{n}+{\left(\text{RB}\right)}_{ln}+{\text{e}}_{iln}$$ (1)

where P_*iln*_ is the observed phenotype of the *i*th variety, the *n*th replicate, and the *l*th sub-block, μ is the general mean of the experiment, g_*i*_ is the *i*th fixed effect of variety, and R_*n*_ is the random effect of the *n*th replicate. (RB)_*ln*_ is the random effect of the *l*th sub-block within the *n*th replicate. e_*iln*_ is the error term. The fixed effect is indicated by a bold lower case letter.

For category II traits, arithmetic means and SDs were calculated. Individual values lying outside the range of 2.5 SD from the arithmetic mean were excluded from further analysis. The total numbers of analyzed plants are documented in Supplementary Table S4. Adjusted means for individual environments were calculated based on Equation 2.

$${\text{P}}_{jin}=\mu +g_{i}+n_{j}+{\left(gn\right)}_{ij}+{\left(\text{NR}\right)}_{jn}+{\text{e}}_{jin}$$ (2)

where P_*jin*_ is the observed phenotype of the *i*th variety, in the *j*th main block, and the *n*th replicate, μ is the general mean of the experiment, g_*i*_ is the *i*th fixed effect of variety, and n_*j*_ the fixed effect of the *j*th N fertilization level, (gn)_*ij*_ is the interaction of the *i*th genotype with the *j*th N fertilization level, and (NR)_*jn*_ is the random effect of the *n*th replicate within the *j*th main block. e_*jin*_ is the error term. Fixed effects are indicated by bold lower case letters.

Secondly, the model described in Equation 3 was used to calculate means across all environments based on adjusted means of individual environments. The same model was also used for ANOVA.

$${\text{P}}_{ijkm}=\mu +g_{i}+n_{j}+{\left(gn\right)}_{ij}+{\text{Y}}_{k}+{\left(\text{YL}\right)}_{km}+{\text{e}}_{ijkm}$$ (3)

where P_*ijkm*_ is the observed phenotype of the *i*th variety, in the *j*th N fertilization level, at the *k*th year and the *m*th location, μ is the general mean of the experiment, g_*i*_ is the *i*th fixed effect of variety, n_*j*_ is the fixed effect of the *j*th N fertilization level, gn_*ij*_ is the interaction of the *i*th genotype with the *j*th N fertilization level, Y_*k*_ is the random effect of the *k*th year, (YL)_*km*_ is the effect of the *m*th location in the *k*th year, and e_*ijkm*_ is the error term. Fixed effects are indicated by the bold lower case letters.

Broad sense heritability was estimated as described in Equation 4, following the concept of equation 19 in Piepho and Möhring (2007).

$$h^{2}=\frac{\;\sigma \text{​}{\;}_{\text{G}}^{2}}{\;\sigma \text{​}{\;}_{\text{G}}^{2}+\;\;\text{​}{\text{ SE}}^{2}}$$ (4)

where $\;\sigma \text{​}{\;}_{\text{G}}^{2}$ is the genetic variance derived from a full random model (Equation 5), SE^2^ is the squared standard error of the difference between the means (derived from Equation 6).

$${\text{P}}_{ijklmn}=\mu +{\text{G}}_{i}+{\text{N}}_{j}+{\left(\text{GN}\right)}_{ij}+{\text{Y}}_{k}+{\text{ L}}_{m}+{\left(\text{YL}\right)}_{km}+{\left(\text{GYL}\right)}_{ikm}+{\left(\text{YLNR}\right)}_{jkmn}+{\left(\text{YLNRB}\right)}_{jklmn}+{\text{e}}_{ijklmn}$$ (5)

where P_*ijklmn*_ is the observed phenotype of the *i*th variety, in the *j*th N fertilization level, in the *k*th year, at the *m*th location, in the *n*th replication, and in the *l*th sub-block, μ is the general mean of the experiment, G_i_ is the *i*th fixed effect of variety, N_*j*_ is the fixed effect of the *j*th N fertilization level, (GN)_*ij*_ is the interaction of the *i*th genotype with the *j*th N fertilization level, Y_*k*_ is the effect of the *k*th year, L_*m*_ is the effect of the *m*th location, (YL)_*km*_ is the interaction effect of the *mt*h location within the *k*th year, (GYL)_*ikm*_ is the interaction of the *i*th genotype with the *mt*h location and the *k*th year, (YLNR)_*jkmn*_ is the effect of the *n*th replication, within the *j*th N fertilization level, within the *m*th location, within the *k*th year, (YLNRB)_*jklmn*_ is the effect of the *l*th sub-block within the *n*th replication, within the *j*th N fertilization level, within the *m*th location, within the *k*th year, and e_*ijklmn*_ is the error term.

$${\text{P}}_{ijkm}=\mu +g_{i}+n_{j}+y_{k}+l_{m}+{\left(yl\right)}_{km}+\;{\left(\text{GN}\right)}_{ij}+{\left(\text{GYL}\right)}_{ikm}+{\text{ e}}_{ijkm}$$ (6)

where P_*ijkm*_ is the phenotype of the *i*th variety, in the *j*th N fertilization level, in the *k*th year, at the *m*th location, μ is the general mean of the experiment, g_*i*_ is the *i*th fixed effect of variety, and n_*j*_ the fixed effect of the *j*th N fertilization level, Y_*k*_ is the fix effect of the *k*th year, l_*m*_ is the fix effect of the *m*th location, (yl)_*km*_ is the interaction of the *m*th location within the *k*th year, (GN)_*ij*_ is the random interaction term of the *i*th genotype with the *j*th N fertilization level, (GYL)_*ikm*_ is the random interaction term of the *i*th genotype with the *m*th location and the *k*th year, and e_*ijkm*_ is the error term. Fixed effects are indicated by bold lower case letters.

All statistical analyses were carried out with the software R (R Core Team, 2013) using the packages lmerTest (Kuznetsova *et al.*, 2016), lsmeans (Lenth, 2016), and lme4 (Bates *et al.*, 2015*a*, b). Pearson product moment correlation was based on all tested varieties (*n*=30). To test whether differences in Nup influence NutE and thus change NUE, the contribution of direct and indirect effects of Nup to overall NUE were analyzed in a path analysis using the R package ‘lavaan’ (Rosseel, 2012). The indirect effects of the path coefficients of Nup on NutE and NutE on NUE were considered together with the direct effect of Nup on NUE. Values of Nup, NutE, and NUE were standardized by *z*-transformation before path analysis.

## Results

### Nitrogen uptake until flowering

Plant biomass production was determined at the flowering peak of MRs (BBCH 67–69). Adjusted genotypic values across two locations (MOS and RHH) in 2 years (2015 and 2016) indicate significant phenotypic variation of 1130 g m^–2^ fresh mass and 165 g m^–2^ dry mass at low N. At high N, the corresponding variation was 1102 g m^–2^ for fresh mass and 155 g m^–2^ for dry mass. The arithmetic means across all genotypes for fresh mass were 4641 g m^–2^ at low N and 5261 g m^–2^ at high N, compared with 733 g m^–2^ at low N and 742 g m^–2^ at high N for dry mass. Fresh mass showed a highly significant difference between low N and high N (*P*-value <0.001), whereas no significant differences were observed between low N and high N for dry mass (Table 1).

**Table 1.** Descriptive statistics and ANOVA of category I traits under low and high nitrogen fertilization levels

Trait	Unit	Low nitrogen fertilization					High nitrogen fertilization					LN/HN	*R* ^2*a*^	Variety	N	Variety×N
		Min	Max	Mean	Range	CoV	Min	Max	Mean	Range	CoV					
Plant fresh mass	g m^–2^	4032.96	5162.62	4641.06	1129.66	0.06	4742.83	5844.98	5260.78	1102.15	0.05	0.88	0.49	***	†	
Plant dry mass	g m^–2^	634.78	799.51	733.41	164.73	0.05	664.85	819.94	742.26	155.09	0.05	0.99	0.48			
N conc leaves	%	3.40	4.01	3.64	0.61	0.04	4.04	4.91	4.38	0.87	0.05	0.83	0.49	†	†	
C conc leaves	%	41.84	43.37	42.73	1.53	0.01	41.42	43.99	42.83	2.57	0.01	1.00	0.42	†		**
CN ratio leaves		11.29	13.59	12.44	2.30	0.04	7.42	10.80	9.73	3.38	0.07	1.28	0.38		†	
N conc stems	%	1.38	1.64	1.48	0.26	0.04	1.48	2.16	1.88	0.68	0.09	0.79	0.18	†	†	†
C conc stems	%	42.96	43.51	43.24	0.54	0.00	42.03	43.66	42.94	1.63	0.01	1.01	–0.05	**	†	**
CN ratio stems		27.50	32.56	30.41	5.05	0.04	20.79	29.73	24.24	8.94	0.08	1.25	0.24	**	†	
N conc siliques	%	4.54	4.93	4.75	0.39	0.02	4.71	5.41	4.98	0.70	0.03	0.95	0.16		†	
C conc siliques	%	46.68	48.12	47.52	1.44	0.01	47.05	48.39	47.81	1.34	0.01	0.99	0.09	*	†	
CN ratio siliques		9.70	10.50	10.07	0.81	0.02	8.82	10.19	9.67	1.37	0.03	1.04	0.10		†	
Leaves mass	g	7.41	9.44	8.31	2.03	0.07	6.92	15.18	10.33	8.27	0.20	0.80	0.02	*	†	*
Stem mass	g	13.07	22.50	16.72	9.43	0.13	8.61	25.63	18.01	17.02	0.22	0.93	0.13	*		
Siliques mass	g	4.40	5.96	5.10	1.56	0.08	3.63	8.33	5.57	4.71	0.19	0.92	0.27	***	**	*
Leaves proportion		0.26	0.31	0.28	0.04	0.04	0.26	0.35	0.30	0.09	0.07	0.94	0.18	***	†	*
Stem proportion		0.52	0.58	0.56	0.06	0.03	0.47	0.58	0.54	0.11	0.05	1.03	0.37	†	†	
Siliques proportion		0.14	0.20	0.16	0.05	0.07	0.14	0.19	0.16	0.05	0.09	1.03	0.44	**		
Leaves N yield	g	0.27	0.39	0.32	0.11	0.10	0.30	0.75	0.49	0.45	0.23	0.66	0.06	*	†	
Stem N yield	g	0.20	0.38	0.26	0.17	0.15	0.20	0.63	0.36	0.43	0.27	0.73	0.29	**	†	*
Siliques N yield	g	0.21	0.30	0.25	0.10	0.08	0.18	0.43	0.28	0.25	0.21	0.90	0.30	***	***	*
Plant N yield	g	0.75	1.02	0.84	0.27	0.10	0.72	1.66	1.13	0.94	0.21	0.74	0.19	**	†	
Leaves C yield	g	3.12	4.13	3.54	1.01	0.08	3.00	6.20	4.39	3.20	0.19	0.81	0.05	*	†	
Stem C yield	g	5.40	9.67	7.20	4.27	0.13	3.72	10.76	7.50	7.04	0.22	0.96	0.15	*		
Siliques C yield	g	2.06	2.83	2.42	0.78	0.08	1.73	3.95	2.67	2.21	0.19	0.91	0.24	***	***	
Plant C yield	g	11.23	16.13	13.13	4.90	0.09	8.44	19.86	14.51	11.42	0.19	0.90	0.10		**	
Single plant miomass	g	26.22	37.12	30.03	10.90	0.09	19.21	45.64	34.01	26.43	0.19	0.88	0.05		***	
No. of plants	*n* m^–2^	28.62	39.53	32.70	10.90	0.08	24.99	38.73	29.99	13.73	0.11	1.09	0.18		***	
Nup	g m^–2^	17.47	21.19	19.62	3.72	0.04	15.39	24.42	20.27	9.03	0.11	0.97	–0.19			
NutE	g g^–1^	24.94	29.62	27.44	4.68	0.04	14.49	26.18	19.89	11.69	0.14	1.38	0.02		†	

Mean is expressed as the arithmetic mean of all tested genotypes.

^*a*^ *R* ^2^ indicates the Pearson coefficient of correlation between low and high nitrogen fertilization.

CoV, coefficient of variation.

*Significantly different from zero at the 0.5 level of probability; **significantly different from zero at the 0.1 level of probability; ***significantly different from zero at the 0.01 level of probability; †significantly different from zero at the 0.001 level of probability.

Although the leaf C concentration was not significantly different between low N and high N (42.73% versus 42.83%), significant variation caused by the genotype for leaf concentration of both N and C was determined within both N fertilization levels. Variation for N in leaves ranged from 3.40% to 4.01% at low N (*P*-value <0.001) and from 4.04% to 4.91% at high N (*P*-value <0.001). As a consequence, the arithmetic mean of C/N ratios of leaves was also shifted towards a higher value at low N (12.44 at low N and 9.73 at high N). N concentration as well as the stem C/N ratio of leaves showed highly significant differences (*P*-value <0.001) between N fertilization levels. While N concentration and the C/N ratio in stems also showed significant differences (*P*-value <0.001) between N fertilization levels and between genotypes, the N concentration of siliques differed only between N fertilization levels but not between genotypes (Table 1).

**Fig. 1.**
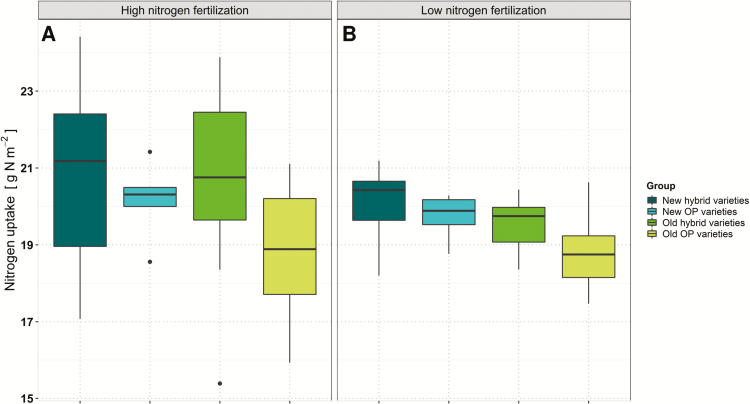
Average adjusted means of nitrogen uptake until flowering for individual variety groups across four environments at (A) high and (B) low nitrogen fertilization. Data were calculated by multiplying the plant dry mass per plot area by the average nitrogen concentration of tested plants.

Multiplication of plant dry mass measured on the plot level by N concentrations revealed the Nup until BBCH 69 for individual genotypes. Nup values varied from 17.47 g N m^–2^ to 21.19 g N m^–2^, with an arithmetic mean of 19.62 g N m^–2^ at low N. At high N, Nup ranged from 15.39 g N m^–2^ to 24.42 g N m^–2^, with an arithmetic mean of 20.27 g N m^–2^. As indicated in Fig. 1, hybrid varieties take up more N until flowering on average than genotypes belonging to OP varieties, especially at high N. At both N fertilization levels, old OP varieties showed the lowest Nup.

NutE was calculated according to Moll *et al.* (1982) as the ratio of seed yield divided by Nup. At low N, the NutE ranged from 24.94 to 29.62 with an arithmetic mean of 27.44. At high N, the NUtE ranged from 14.49 to 26.18 with an arithmetic mean of 19.89. For vegetative traits, we found significant variety×N fertilization level interaction only for the N concentration in stems (*P*-value 0.001) and C concentration in leaves and stems (*P*-value 0.1, Table 1).

The highest heritability was determined for C and N (both: *h*^2^=0.62) concentration in leaves, plant fresh mass (*h*^2^=0.64) and dry mass (*h*^2^=0.55), followed by the C/N ratio in leaves (*h*^2^=0.50) as well as the relative proportion of stem mass to the entire plant biomass (*h*^2^=0.40, Table 2).

**Table 2.** Genotypic variance and interaction with environmental effects according to the mixed linear model (Equation 5) with all factors considered as random for category I traits

Trait	Genotype		Genotype:N		Genotype:Year:Loc		N		Year		Loc		Year:Loc		Year:Loc:N:Rep		Year:Loc:N:Rep:Blk		Residual	*h* ^2^
	Variance	*P*-value	Variance	*P*-value	Variance	*P*-value	Variance	*P*-value	Variance	*P*-value	Variance	*P*-value	Variance	*P*-value	Variance	*P*-value	Variance	*P*-value	Variance	
Plant fresh mass	37 317.0000	†	0.0000		44 714.0000	†	220 730.0000	***	0.0009		369 400.0000		115 130.0000		155 490.0000	†	96 466.0000	†	109 080.0000	0.64
Plant dry mass	663.7400	***	0.0000		777.7100	***	139.7500		1028.6800		9711.3500		287.5000		4298.5500	†	1422.9200	†	5783.6600	0.55
N conc leaves	0.0119	***	0.0023		0.0053	*	0.2735	†	0.0000		1.0688	*	0.0918	***	0.0211	†	0.0096	**	0.0548	0.62
C conc leaves	0.1037	***	0.0272		0.0006		0.0000		0.0000		0.0892		0.1114	†	0.0000		0.0576	*	0.5066	0.62
C/N ratio leaves	0.0968		0.0279		0.1132	***	4.4398	†	0.0000		10.1570		2.1562	***	0.5093	†	0.1752	***	0.7086	0.50
N conc stems	0.0001		0.0027	**	0.0039	†	0.0868	†	0.0214		0.0785	**	0.0000		0.0055	†	0.0024	*	0.0204	0.01
C conc stems	0.0000		0.0286	**	0.0120		0.0227		0.0247		0.1182		0.1398	***	0.0105	*	0.0253		0.2484	0.00
C/N ratio stems	0.0293		0.2369		1.8508	†	24.0079	†	10.5558		32.2251	*	0.1481		3.0809	†	0.4154		6.5925	0.03
N conc siliques	0.0009		0.0032		0.0101	†	0.0465	**	0.1180	*	0.0965	*	0.0000		0.0197	†	0.0047		0.0447	0.12
C conc siliques	0.0201		0.0117		0.0000		0.0218		0.0000		0.6041		0.3056	†	0.0062		0.0000		0.5752	0.32
C/N ratio siliques	0.0000		0.0095		0.0402	†	0.1488	*	0.4682	*	0.2457		0.0000		0.0827	†	0.0250	**	0.1597	0.00
Leaves mass	0.0900		0.0000		0.1577		1.2551		0.0000		0.0000		35.8300	†	0.9242	†	0.0000		4.3953	0.20
Stem mass	1.0068		0.0000		2.8164	*	0.3441		12.8051		0.0000		120.8481	†	0.6808		0.0000		33.6470	0.25
Siliques mass	0.0190		0.0000		0.2099	***	0.0646		0.0000		0.0000		17.6880	†	0.0167		0.1120		1.1211	0.12
Leaves proportion	0.0000		0.0000		0.0000		0.0001	**	0.0000		0.0002	*	0.0000		0.0000	*	0.0001		0.0009	0.31
Stem proportion	0.0001		0.0000		0.0002	**	0.0001	**	0.0000		0.0000		0.0014	†	0.0000		0.0002	**	0.0012	0.40
Siliques proportion	0.0000		0.0000		0.0001	***	0.0000		0.0000		0.0000		0.0017	†	0.0001	†	0.0001	**	0.0006	0.31
Leaves N yield	0.0001		0.0003		0.0008		0.0091	**	0.0000		0.0131		0.0891	†	0.0036	†	0.0000		0.0097	0.09
Stem N yield	0.0005		0.0000		0.0015	**	0.0039	***	0.0021		0.0000		0.0535	†	0.0006	**	0.0000		0.0119	0.28
Siliques N yield	0.0001		0.0000		0.0005	***	0.0003	**	0.0000		0.0000		0.0522	†	0.0000		0.0004	**	0.0030	0.19
Plant N yield	0.0010		0.0002		0.0072	**	0.0308	**	0.0000		0.0000		0.5862	†	0.0093	†	0.0000		0.0553	0.13
Leaves C yield	0.0192		0.0000		0.0268		0.2217		0.0000		0.0000		6.5876	†	0.1784	†	0.0000		0.8042	0.22
Stem C yield	0.2143		0.0000		0.5119	*	0.0000		2.7232		0.0000		20.9730	†	0.1231		0.0000		6.2202	0.27
Siliques C yield	0.0042		0.0000		0.0487	***	0.0156		0.0000		0.0000		4.0823	†	0.0065		0.0247		0.2590	0.11
Plant C yield	0.1746		0.0000		1.1438	*	0.5538		0.0000		0.0000		85.6960	†	0.9287	***	0.0000		12.6860	0.13
Single pl. biomass	0.5330		0.0000		5.9742	*	4.5987		0.0000		0.0000		455.7200	†	4.9374	***	0.0000		67.0560	0.08
No. of plants	1.2144		0.0000		2.5998		1.6298		39.5840		0.0000		240.1900	†	4.5617	***	5.3137		56.7330	0.20

*Significantly different from zero at the 0.5 level of probability; **significantly different from zero at the 0.1 level of probability; ***significantly different from zero at the 0.01 level of probability; †significantly different from zero at the 0.001 level of probability.

### Flowering time

**Fig. 2.**
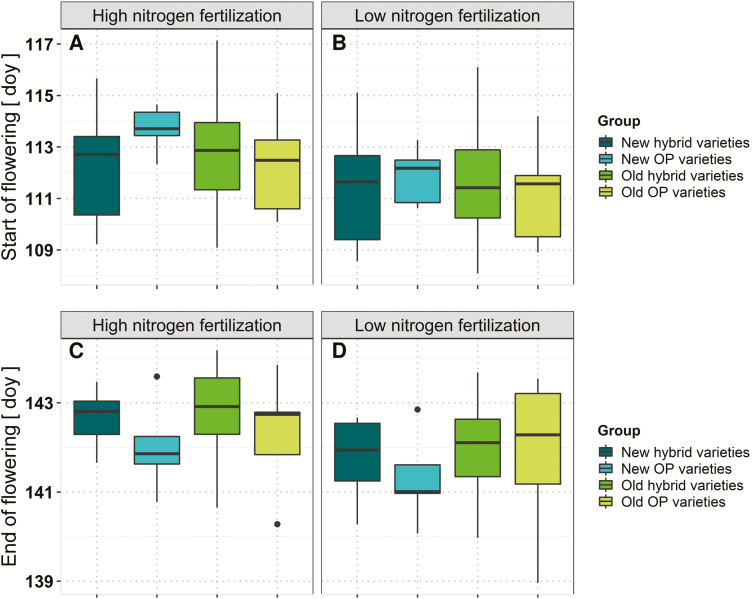
Average adjusted means of number of days of the year (doy) until (A, B) the start and (C, D) the end of flowering for individual variety groups determined over six environments at (A, C) high and (B, D) low nitrogen fertilization.

Start of flowering, measured in doy, ranged at low N from 108 to 116 doy with a mean of 112. End of flowering varied between 139 and 144 doy, with an average of 142. The start and end of flowering were highly affected by the genotype and N fertilization level (Table 3). As indicated in Fig. 2, new hybrid varieties and old OP varieties tended towards an earlier onset of flowering. However, new OP varieties tested in this study generally finished flowering earlier than members of the other groups and therefore had a shorter flowering duration.

**Table 3.** Descriptive statistics and ANOVA of category II traits under low and high nitrogen fertilization levels

Trait	Unit	Low nitrogen fertilization					High nitrogen fertilization					LN/HN	*R* ^2*a*^	Variety	N	Variety×N
		Min	Max	Mean	Range	CoV	Min	Max	Mean	Range	CoV					
Number of SBs	*n*	5.89	9.54	7.71	3.65	0.13	6.11	9.27	7.67	3.16	0.11	1.01	0.63	†		
TSW	g	4.02	5.08	4.61	1.06	0.05	4.02	5.24	4.61	1.22	0.06	1.00	0.95	†		
Number of siliques MR	*n*	33.61	62.56	49.97	28.94	0.12	32.98	62.40	51.87	29.42	0.14	0.96	0.87	†	**	
No. of seeds per silique MR	*n*	9.68	18.31	14.63	8.63	0.13	9.76	15.93	13.26	6.17	0.12	1.10	0.45	†	†	
Yield MR	g	2.07	4.31	3.27	2.24	0.17	1.61	4.11	3.08	2.50	0.19	1.06	0.71	†	*	
No, of siliques SB	*n*	109.56	251.11	179.97	141.56	0.20	146.28	241.22	197.21	94.94	0.13	0.91	0.27	*	**	
No. of seeds per silique SBs	*n*	6.93	13.90	10.63	6.97	0.13	6.32	12.50	9.95	6.18	0.12	1.07	0.35	**	**	
Yield SB	g	4.75	12.24	8.68	7.49	0.21	5.78	11.65	8.93	5.87	0.17	0.97	0.37	*		
Seed yield	t ha^–1^	3.96	5.18	4.71	1.21	0.05	4.00	5.27	4.84	1.27	0.07	0.97	0.91	†	†	
Oil concentration	%	44.02	47.60	45.75	3.59	0.02	41.47	45.87	44.04	4.41	0.02	1.04	0.97	†	†	
Protein concentration	%	14.54	16.37	15.37	1.83	0.03	16.37	18.49	17.26	2.12	0.04	0.89	0.94	†	†	
Start of flowering	doy	108.08	116.10	111.58	8.02	0.02	109.09	117.14	112.74	8.05	0.02	0.99	0.95	†	†	
End of flowering	doy	138.96	143.68	141.80	4.72	0.01	140.28	144.18	142.50	3.90	0.01	1.00	0.93	†	†	
Flowering duration	no. of days	27.56	32.81	30.23	5.25	0.05	26.19	33.15	29.76	6.96	0.05	1.02	0.92	†	**	
Plant length	cm	134.72	166.97	147.83	32.25	0.05	133.89	176.21	154.53	42.32	0.05	0.96	0.96	†	†	
NUE	ratio	33.05	43.13	39.26	10.09	0.05	18.20	23.96	22.00	5.76	0.07	1.78	0.91	†	†	

Mean is expressed as the arithmetic mean of all tested genotypes.

^*a*^ *R* ^2^ indicates the Pearson coefficient of correlation between low and high nitrogen fertilization.

MR, main raceme; SB, side branch; CoV, coefficient of variation

*Significantly different from zero at the 0.5 level of probability; **significantly different from zero at the 0.1 level of probability; †significantly different from zero at the 0.001 level of probability.

### Plant length

**Fig. 3.**
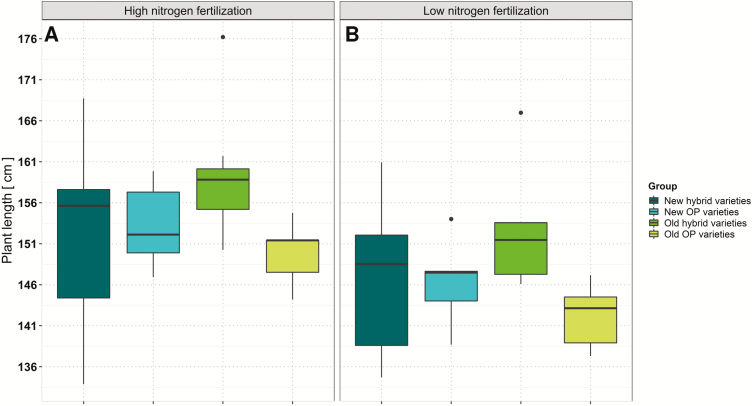
Average adjusted means of plant lengths for individual variety groups determined over six environments at (A) high and (B) low nitrogen fertilization.

Plant length, significantly affected by variety and N fertilization level, varied at low N between 135 cm and 167 cm with an arithmetic mean of 148 cm. At high N, it varied from 134 cm to 176 cm with an arithmetic mean of 155 cm. As indicated in Fig. 3, hybrid varieties tended to achieve a higher plant canopy than OP varieties, with the difference being particularly noticeable between older hybrids and older OP varieties.

### Seed yield and primary yield components

**Fig. 4.**
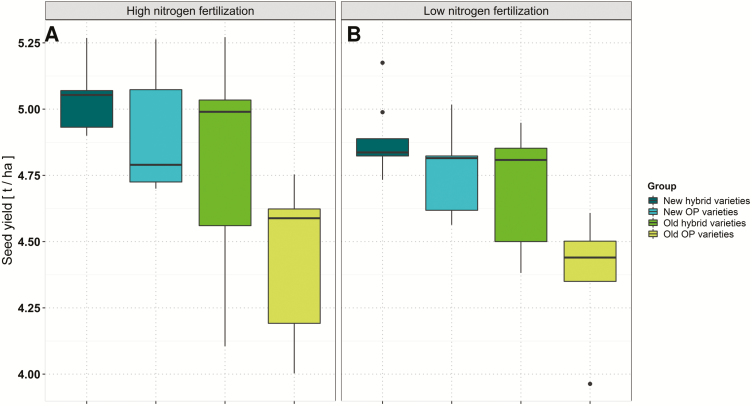
Average adjusted means of seed yield for individual variety groups determined over six environments at (A) high and (B) low nitrogen fertilization.

**Fig. 5.**
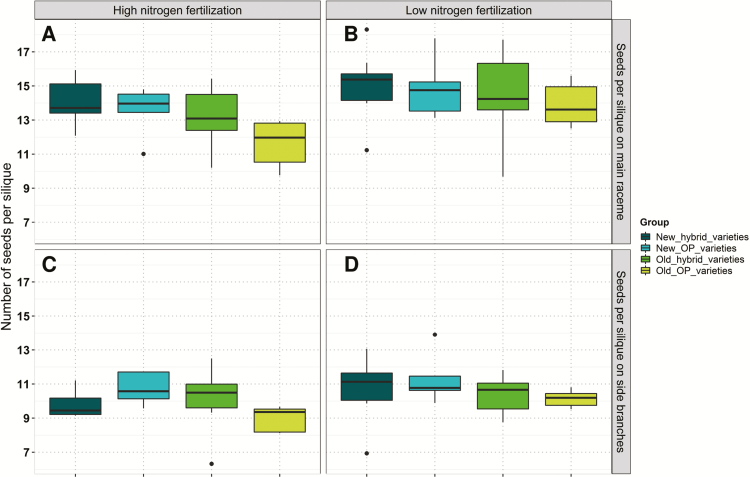
Average adjusted means of numbers of seeds per silique on (A, B) the main raceme and (C, D) side branches for individual variety groups determined over six environments at (A, C) high and (B, D) low nitrogen fertilization.

Seed yield (measured on the plot level) was highly significantly affected by the variety and N fertilization but not by the interaction between these two factors. Seed yield varied from 3.96 t ha^–1^ to 5.18 t ha^–1^ at low N and from 4.00 t ha^–1^ to 5.27 t ha^–1^ at high N (Table 3). At both fertilization levels, modern hybrid varieties outperformed the other variety groups on average. Old OP varieties showed the lowest seed yield by far of all variety groups (Fig. 4). Measurement of YCs was done shortly before machine harvest of the plots. All traits measured on the MR showed highly significant genotype effects. ANOVA for primary YC determination revealed that seed yield of individual plant segments (MR and SRs), number of SBs per plant, and TSW do not differ between the two N fertilization levels. Only the number of siliques (*P*-value <0.1) and the number of seeds per silique showed significant differences between high N and low N on both the MR (*P*-value <0.001)and SBs (*P*-value <0.1,Table 3). On the MR, an average of 14.63 and 13.26 seeds per silique were determined at low and high N, respectively. On the SBs, the average number of seeds per silique was 10.63 at low N and 9.95 at high N. Interestingly, this corresponds to an average of 1.37 more seeds per silique at low N compared with high N for the MR (Table 3). For both the MR and SBs, both the number of siliques (MR, *r*=0.66, *P*-value <0.001; SBs, *r*=0.65, *P*-value <0.001 for low N; and MR, *r*=0.77, *P*-value <0.001; SBs, *r*=0.55, *P*-value 0.001 at high N) and the number of seeds per silique (MR, *r*=0.67, *P*-value <0.001; SBs, *r*=0.39, *P*-value 0.034 for low N; and MR, *r*=0.79, *P*-value <0.001; SBs, *r*=0.66, *P*-value <0.001 for high N) were positively correlated with seed yield of individual plant segments, confirming that these two traits both contribute to higher yields. Furthermore, the ANOVA revealed no significant G×N interactions in this study (Table 3). Separation of all tested varieties into groups indicated that modern varieties, regardless of whether they are hybrid or OP varieties, outperform older varieties in terms of their average number of seeds per silique on the MR, at both N levels. The same was true for the SB under the low N treatment. At high N, the new OP and old hybrid varieties showed the highest average values (Fig. 5).

While TSW was not significantly influenced by the N fertilization level (at both N fertilization levels: 4.61 g), the varietal effect on TSW was highly significant. At low N, it ranged from 4.02 g to 5.08 g and at high N from 4.02 g to 5.24 g (Table 3; Supplementary Fig. S1).

**Fig. 6.**
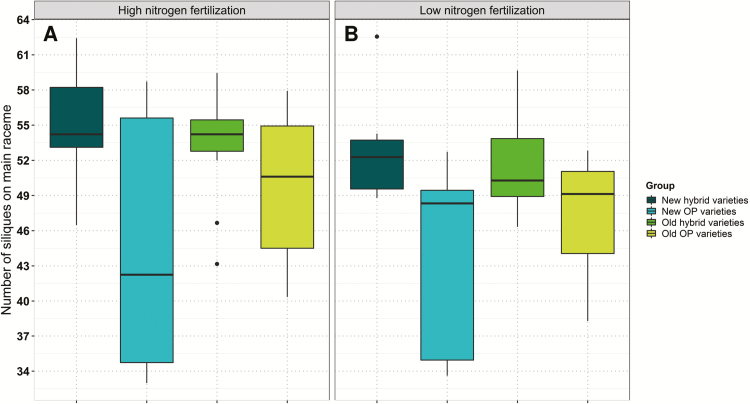
Average adjusted means of number of siliques on the main raceme for individual variety groups determined over six environments at (A) high and (B) low nitrogen fertilization.

**Fig. 7.**
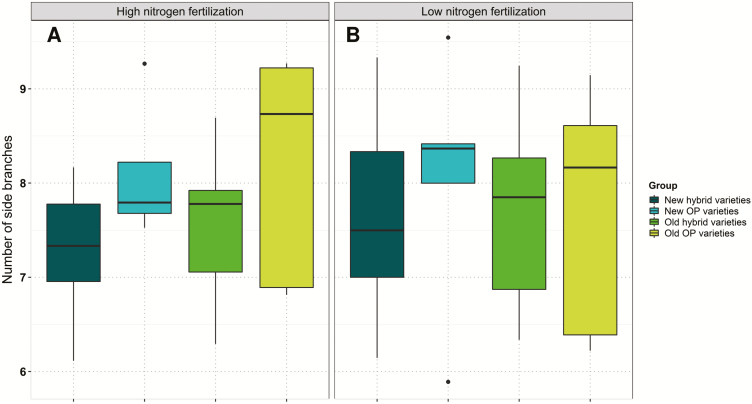
Average adjusted means of number of side branches for individual variety groups determined over six environments at (A) high and (B) low nitrogen fertilization.

The number of siliques on the main raceme showed a range of 28.94 at low N and 29.42 at high N. Comparisons between OP and hybrid varieties revealed that hybrids tend to bear more siliques on the MR (Fig. 6) but not on the SBs. There, under high N, older varieties showed a higher number of siliques per MR than modern varieties, whereas no significant difference was seen in number of siliques per SB (Supplementary Fig. S3). The number of SBs per plant was significantly influenced by the genotype, ranging from 5.89 to 9.54 (mean 7.71) at low N and from 6.11 to 9.27 (mean 7.67) at high N. New hybrid varieties had fewer branches on average at both N fertilization levels (Fig. 7).

Vegetative traits had low heritability values. In contrast, category II traits were higher (Tables 2, 4). The highest heritability was determined for TSW and seed oil concentration (both *h*^2^=0.93), and start of flowering (*h*^2^=0.91) and seed protein concentration (*h*^2^=0.90). Notably, heritabilities for primary YCs on the SBs are characterized by a lower heritability than on the MR (Table 4).

**Table 4.** Genotypic variance and interaction with environmental effects according to the mixed linear model (Equation 5) with all factors considered as random for category II traits

Trait	Genotype		Genotype:N		Genotype:Year:Loc		N		Year		Loc		Year:Loc		Year:Loc:N:Rep		Year:Loc:N:Rep:Blk		Residual	Heritability
	Variance	*P*-value	Variance	*P*-value	Variance	*P*-value	Variance	*P*-value	Variance	*P*-value	Variance	*P*-value	Variance	*P*-value	Variance	*P*-value	Variance	*P*-value	Variance	
Number of SBs	0.4151	***	0.0345		0.5055	†	0.0000		1.4425	*	0.0052		0.2199		0.4710	†	NA	NA	5.1447	0.63
TSW	0.0557	†	0.0012	*	0.0171	†	0.0000		0.0000		0.0084		0.0238	†	0.0063	†	NA	NA	0.0381	0.93
Number of siliques on MR	34.5005	†	0.0000		22.5753	†	1.1075		0.0000		50.9179		15.7598	***	4.9911	***	NA	NA	133.4201	0.81
No. of seeds per silique on MR	1.0128		0.4389		2.3330	†	0.9908	***	0.0000		10.2536		4.7532	†	0.4534	**	NA	NA	17.2714	0.47
Yield on MR	0.1981	†	0.0000		0.2098	†	0.0166		0.0000		0.1608		0.3557	†	0.0085		NA	NA	1.4524	0.71
Number of siliques on SBs	300.3050		59.1280		492.0800	**	81.6860		896.3170		444.0850		944.3870	**	754.9990	†	NA	NA	9542.5330	0.44
No. of seeds per silique on SBs	0.3901		0.1233		0.7834	**	0.1991		0.0000		9.2322		6.4405	†	0.5400	***	NA	NA	14.2289	0.38
Yield on SB	0.8328	**	0.0000		0.8085		0.0000		0.0000		1.5295		10.2470	†	1.8687	†	NA	NA	32.4510	0.42
Seed yield on plot level	0.0692	†	0.0048	***	0.0466	†	0.0076	**	0.0000		0.4014	***	0.0017		0.0076	†	0.0129	†	0.0835	0.85
Seed protein concentration	0.2865	†	0.0075		0.1074	†	1.7762	†	0.0310		0.7334		1.1138	†	0.0993	†	0.1335	†	0.2536	0.90
Seed oil concentration	0.9328	†	0.0218	**	0.2556	†	1.4537	†	0.0000		0.5986		0.9300	†	0.1665	†	0.0799	†	0.5122	0.93
Start of flowering	4.0430	†	0.0034		1.3724	†	0.6612	***	48.6600	*	10.0369		0.7896	*	0.4773	†	0.5330	†	0.7400	0.91
End of flowering	1.2847	†	0.0131		0.7429	†	0.4135	†	0.8646	*	4.7651	**	0.0000		0.0563	***	0.1011	**	0.6805	0.85
Flowering duration	2.4763	†	0.0000		1.6874	†	0.0000		36.3072	**	0.4844		1.0893	**	0.4059	†	0.2610	†	1.3103	0.83
Plant length	58.8249	†	0.3690		16.6790	†	10.4161	**	0.0000		312.2605		73.6935	†	13.2528	†	13.5039	†	22.9697	0.83

*Significantly different from zero at the 0.5 level of probability; **significantly different from zero at the 0.1 level of probability; ***significantly different from zero at the 0.01 level of probability; †significantly different from zero at the 0.001 level of probability.

### Trait inter-relationships

**Fig. 8.**
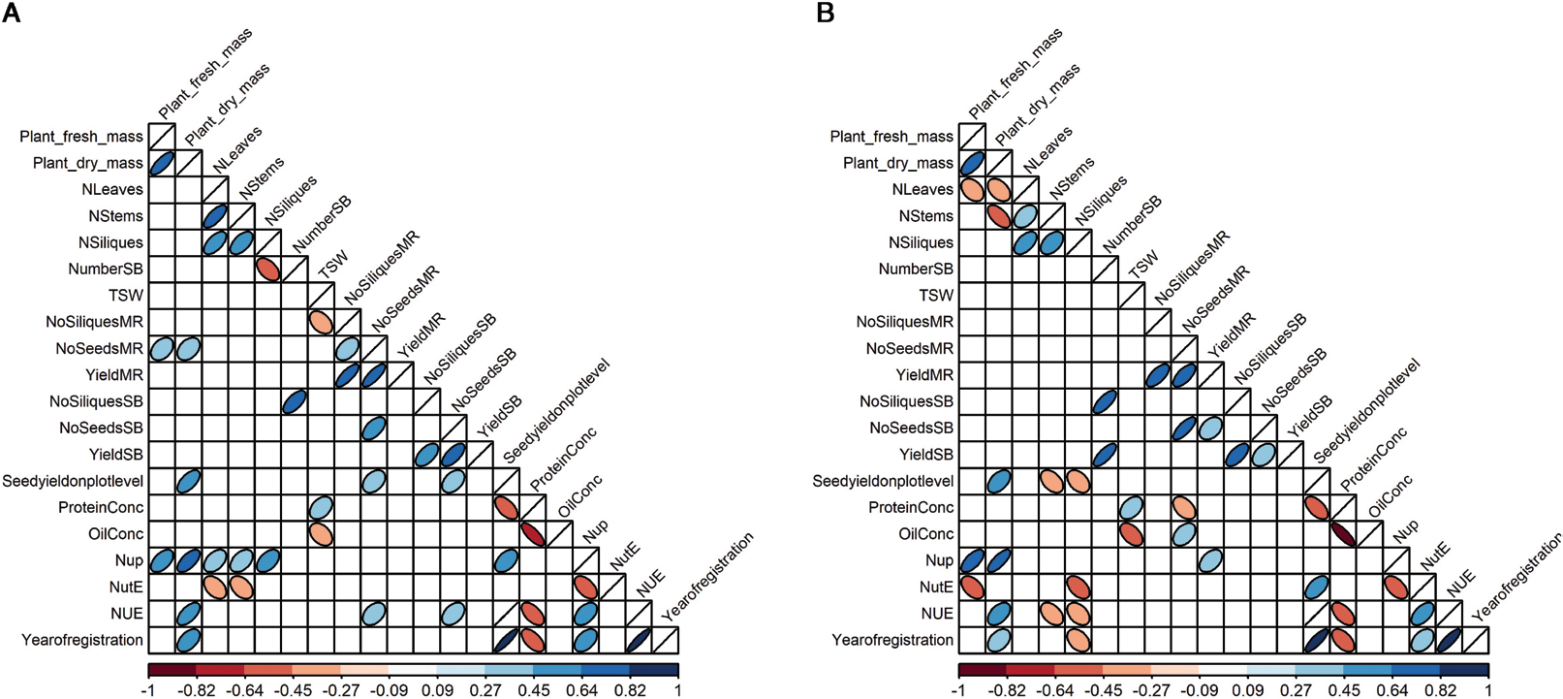
Intertrait correlations among most relevant traits at (A) high and (B) low nitrogen fertilization. Direction and strength of Pearson’s coefficient of correlation are depicted by the shape and depth of shading of the ellipses. Non-significant correlations are shown as white boxes.

To examine the link between observed phenotypic variation attributable to the varieties, with regard to growth parameters and traits relevant for seed yield at low N, pairwise correlations were calculated based on adjusted means (Fig. 8; Supplementary Fig. 4). As expected, fresh plant biomass at flowering under low N correlated significantly (*r*=0.69, *P*-value <0.001) with dry mass, with start (*r*=0.71, *P*-value <0.001) and end of flowering (*r*=0.57, *P*-value 0.001), and with plant length (*r*=0.44; *P*-value 0.014); however, fresh plant biomass correlated negatively with duration of flowering (*r*= –0.51, *P*-value 0.004). Although fresh mass was not correlated with seed yield, dry mass showed a significant relationship to seed yield (*r*=0.48, *P*-value 0.010). Plant length was positively correlated with seed yield at low N (*r*=0.38, *P*-value 0.038) but not at high N.

The N concentrations in leaves, stems, and siliques were all positively correlated with each other, meaning that genotypes superior in N concentration in one plant partition are also superior in N concentration of both other plant partitions. However, whereas the N concentration in leaves at flowering was positively correlated with seed oil concentration (*r*=0.36, *P*-value 0.050), this was not the case for N concentration in stems and siliques. Instead these two traits showed negative correlations with seed yield (*r*= –0.44, *P*-value 0.015 for stems and *r*= –0.40, *P*-value 0.028 for siliques).

The number of SBs correlated with the cumulative number of siliques on the SBs (*r*=0.72, *P*-value <0.001) at low N and (*r*=0.64, *P*-value <0.001) at high N. The number of SBs showed a stronger positive correlation with seed yield on the SBs at low N (*r*=0.75, *P*-value <0.001) than at high N (*r*=0.35, *P*-value 0.059).

Only at high N was a correlation observed between the seed yield measured in plots and the number of seeds per silique on the MR (*r*=0.38, *P*-value 0.037) and SBs (*r*=0.41, *P*-value 0.025). In contrast, no significant correlation was found between the number of siliques on the side branches and the seed yield of plots under high N. At low N, correlations between number of seeds per silique and plot seed yield were *r*=0.29 (*P*-value 0.114) for the MR and *r*=0.24 (*P*-value 0.197) for SBs at low N. Although these correlations were not significant, they were greater than the correlations of any other YC to seed yield.

The number of seeds per silique on the main raceme was positively correlated with seed oil concentration at low N (*r*=0.42, *P*-value 0.022) but not at high N (*r*=0.15, *P*-value 0.436). The number of seeds per silique and the number of siliques showed positive relationships to seed yield of particular plant segments at both N levels on both the MR and SBs. Correlation between start and end of flowering were *r*=0.70 (*P*-value <0.001) at low N and of *r*=0.55 (*P*-value 0.002) at high N, indicating that the genotypes differ in flowering duration (Figs 2, 8). End of flowering correlated negatively with number of seeds per silique on SBs at both N levels (low N, *r*= –0.39), *P*-value 0.036; high N, *r*= –0.32, *P*-value 0.084). This relationship was not found for start of flowering.

At both N fertilization levels, no significant correlation was detected between TSW and seed yield; however, TSW was significantly negatively correlated with seed oil concentration (low N, *r*= –0.49, *P*-value 0.006; high N, *r*= –0.39, *P*-value 0.035) and end of flowering (low N, *r*= –0.43., *P*-value 0.018. high N, *r*= –0.44, *P*-value 0.014). Furthermore, exclusively for high N, the number of siliques on the main raceme showed a negative relationship to TSW (*r*= –0.41, *P*-value 0.024).

At low N, plant dry mass at flowering correlated with the number of seeds per silique (*r*=0.32, *P*-value 0.08 on the MR; and *r*=0.33, *P*-value 0.076 on the SBs), while the number of seeds per silique also correlated positively with the proportion of the stems (*r*=0.51, *P*-value 0.004 for the MR; *r*=0.61, *P*-value <0.001 for the SBs) and negatively with the proportion of silique dry mass at flowering (*r*= –0.49, *P*-value 0.006 on the MR; *r*= –0.60, *P*-value <0.001 on the SBs). At the same time, the start and duration of flowering were not associated with the number of seeds per silique. End of flowering correlated only with the number of seeds on the SBs (*r*= –0.39, *P*-value 0.036).

Interestingly, Nup was only correlated with NUE (*r*=0.47, *P*-value 0.009) and with year of registration (*r*=0.46; *P*-value 0.010) under high N but not under low N. Under low N, a much stronger correlation of NutE with NUE (*r*=0.54, *P*-value 0.002) than of Nup with NUE (*r*=0.21; *P*-value 0.249) underlined the greater relevance of NutE for NUE improvement. In path analysis, a negative regression between Nup and NutE (*r*= –0.52 for both N levels) was detected, while both Nup (*r*=0.68 at low N and *r*=0.60 at high N) and NutE (*r*=0.90 at low N and *r*=0.25 at high N) showed a positive effect on NUE.

Finally, canopy dry mass at flowering and NutE were both positively correlated with year of registration at low N (*r*=0.39, *P*-value 0.033 and *r*=0.41, *P*-value 0.026, respectively). At high N, only dry mass (*r*=0.48, *P*-value 0.007) correlated significantly with year of registration, whereas NutE did not. On the other hand, the correlation of seed yield and consequently NUE with the year of registration (*r*=0.83, *P*-value <0.001 at low N and *r*=0.90, *P*-value <0.001 at high N) was considerably stronger than those with any other investigated trait.

## Discussion

Attempts to increase oil and protein yields, while simultaneously reducing environmental damage due to high N fertilization, are urgently required to sustainably achieve food and feed demand of the growing world population. Increasing NUE in oilseed rape, one of the world’s most important dicotyledonous oil and protein crops which does not naturally fix N, therefore has considerable global agro-ecological importance. There is strong evidence in the literature for substantial historical breeding progress enhancing oilseed rape yields under divergent N fertilization levels (Gehringer *et al.*, 2007; Kessel *et al.*, 2012; Stahl *et al.*, 2017). Those gains have resulted in increases of overall yields in major oilseed rape cultivation regions (Kirkegaard *et al.*, 2016), even though these advantages are not always reflected in specific environments (Peltonen-Sainio *et al.*, 2007*a*). In the context of these findings, the present study aims to provide a detailed dissection of growth parameters associated with breeding progress within the last two decades, a period in which global cultivation of oilseed rape expanded vastly across the globe.

### Relevance of Nup and NutE for overall NUE

Harvest of plant biomass at flowering, measurement of flowering time, and determination of N concentration enabled us to distinguish between Nup and NutE. Previous studies pointed out that NutE rather than NupE is the predominant factor influencing NUE under high N. Under low N, however, NupE gains in importance for achieving a high NUE (Berry *et al.*, 2010; reviewed in van Lammerts Bueren and Struik, 2017). In this study, we also found evidence from Pearson product moment and path analysis that at low N NutE is much more important than Nup (*r*=0.90 versus *r*=0.68 in path analysis). However, care must be taken when comparing results across different studies. In the present study, low N is not a zero-N treatment but rather a reduced N input which does not necessarily represent an extreme constraint on seed yield (Stahl *et al.*, 2017). Therefore, the data in our study might be more comparable with reduced (but still high-level) N input levels in many other studies rather than with extremely low N treatments. In contrast, Nup in our high N treatment correlated much more strongly with NUE than at low N. Notably, in line with Gehringer *et al.* (2007) and Wang *et al.* (2016), we also observed a higher Nup and biomass accumulation due to a vigorous vegetative growth of hybrid varieties. Hybrids outperformed OP; however; this was not as prominent at low N (Fig. 1).

In general, the respective positive correlations between plant dry mass at flowering and Nup with the year of registration indicate that direct or indirect selection for pre-flowering traits can partially explain breeding progress for seed yield within the last two decades. In addition, we found that larger genotypes of this study, especially among the hybrids (Fig. 3), are advantageous for seed yield but not for NutE exclusively at low N. This finding is in line with results from He *et al.* (2017). In agreement with other studies (Zhao *et al.*, 2006; Koeslin-Findeklee *et al.*, 2014; Stahl *et al.*, 2016, 2017; He *et al.*, 2017), we found the NutE is negatively correlated with the seed N concentration. This can be explained by a negative correlation between seed oil and protein concentration, along with strong selection during the past two decades towards genotypes with a high oil concentration. Nevertheless, NutE showed a strong association with seed yield.

### Breeding progress associated with changes in primary yield components

Final seed yield is the product of the primary YCs including the number of plants per area, number of siliques per plant, number of seeds per silique, and seed size (e.g. TSW). In light of the fact that N remobilization is rather sink limited under high N input cultivation (Gombert *et al.*, 2010; Ulas *et al.*, 2013), development of YC in relation to N source appears to have a critical relevance. Since improvement of seed yield *per se* is much more important for N yield than seed protein concentration (Stahl *et al.*, 2017), improvement of YC is important to enhance the N offtake from the field, thus lowering the N balance surplus. However, description of yield structure in terms of YC is notoriously difficult in indeterminate dicot crops such as oilseed rape, and the reliability of the description critically depends on whether the data are collected on single plants or at the entire plot level (Diepenbrock, 2000). Despite high residual errors, in our investigations, we consistently observed three findings at both N fertilization levels which therefore seem to represent constitutive effects irrespective of G×N interactions.

First, an increase in SB number led to a higher number of siliques on the SBs and, as a consequence, to a higher seed yield contributed by the SBs. Secondly, a clear advantage of hybrid varieties was found to be their elevated number of siliques. There is strong evidence (see results under ‘Trait inter-relationships’) that higher numbers of siliques on the MR and SBs result in a higher yield from each segment at both N fertilization levels. Furthermore, since modern high-yielding varieties show a lower SB number (Fig. 7), we conclude that most of the modern varieties outperform old OP varieties with higher density of siliques per individual SB (Supplementary Fig. S2). It can be speculated that the superiority of hybrids in terms of the MR rather than the SBs might be due to a higher interaction with neighboring plants. In contrast to the MR, the SBs are more affected by concurrent effects of other branches from the same plant as well as from neighboring plants (Richards and Thurling, 1979; Diepenbrock, 2000). Hence, the higher interactions with environmental factors potentially hide the genetic effect. Further enlargement of the experiment to include more genotypes per group might help to uncover genetic effects. In contrast, the MR is probably less influenced by neighbor effects due to its upright position. These assumptions are supported by contrasting heritability of the same traits between the MR and SBs (Table 4).

Thirdly, varieties released more recently tended to have more seeds per silique. Heritabilities of *h*^2^=0.47 for seeds per silique on the MR and *h*^2^=0.38 on the SBs suggest a breeding-driven modification during the last two decades. This finding is true for both SBs and the MR. Differences in flowering time of the MR and SBs, caused by a delayed flowering of SBs, potentially reduce assimilate transport to siliques on the SBs, is most likely to be the reason for a lower number of seeds per silique on the SBs. However, strong correlation of *r*=0.76 at low N and *r*=0.55 at high N between the MR and SBs suggests that the number of seeds per silique shows a similar varietal effect. However, it remains unclear if the higher number of seeds per silique is achieved by efficient N remobilization or by post-anthesis nutrient uptake. In cereals, an increase in seed yield was also associated with the number of kernels per plant rather than with seed size, and the plasticity of the former depends much more strongly on environmental influence than on TSW (Sadras, 2007; Peltonen-Sainio *et al.*, 2007*b*; Sadras and Slafer, 2012). Zhang *et al.*, (2012) found in rapeseed that the effective number of siliques per plant and the TSW are closely associated with seed yield.

### Stagnation of breeding progress in thousand seed weight and its trade-off to increased seed oil concentration

In contrast to other YCs, no significant trend could be detected for TSW in relation to the year of registration or the type of variety (hybrid versus OP; Supplementary Fig. S1). Earlier studies also implied a lower relevance of TSW for seed yield compared with the effect of the number of seeds per silique and the number of siliques per plant (Scarisbrick *et al.*, 1981; Diepenbrock, 2000; Berry and Spink, 2009; Chen *et al.*, 2014; Labra *et al.*, 2017). We also found no decrease of TSW associated with increasing N fertilization, although such an effect had been observed in a single environment in an older study on spring-type oilseed rape (Hocking *et al.*, 1997).

These results suggest that TSW is not significantly influenced by selection, allowing two possible conclusions: (i) variation in TSW does not contribute to yield improvement and is therefore not influenced by breeding programs focusing solely on yield selection *per se*; or (ii) enhanced TSW might be beneficial for seed yield increase but the effects are masked by other factors influencing seed yield, preventing breeders from actively considering TSW in selection decisions.

**Fig. 9.**
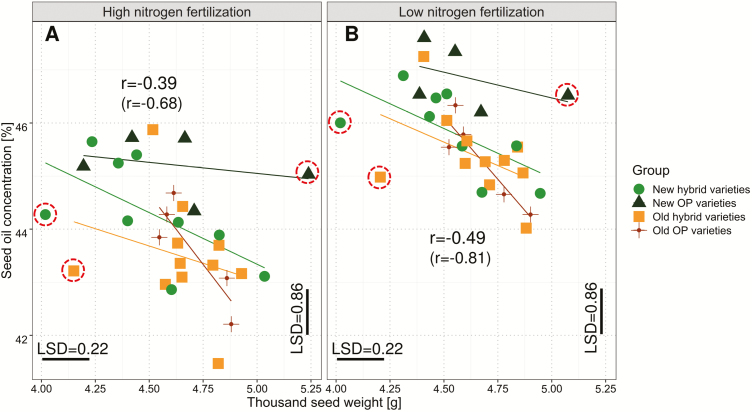
Scatterplot comparing average seed oil concentration versus thousand seed weight for (A) high and (B) low nitrogen fertilization for individual groups of varieties. Data show average values from six independent environments. Trend lines are depicted for each individual group. Correlations (*r*) are shown across all groups. The correlations are lowered by three outlying varieties (dotted circles). Strong negative correlations after excluding those three varieties from the analysis are shown in parentheses.

The latter explanation may also help to clarify the completely unexpected negative correlation between seed oil concentration and TSW. Usually one would expect a positive correlation between TSW and seed oil concentration, as bigger seeds should have a relatively lower proportion of seed hull fraction (fiber) and consequently higher proportions of protein and oil. Indeed, older studies found TSW to be important for plant productivity (Clarke and Simpson, 1978; Butruille *et al.*, 1999; Shi *et al.*, 2009), and TSW was found to be positively associated with oil concentration both in *Brassica juncea* (Singh *et al.*, 1996) and in *B. napus* diversity sets (*r*=0.55; Körber *et al.*, 2016). However, the present studies clearly demonstrate that selection during the past 20 years has completely reversed this relationship. Economic interest in seed oil led breeders towards selection for increased oil concentration (Stahl *et al.*, 2017), whereas TSW remained masked by the seed yield *per se*. The negative correlation between these two traits is pronounced at low N fertilization levels (Fig. 9), but is considerably weaker at high N (the level where selections decisions were made in most breeding programs during the past decades); this is the most probable explanation for why this negative relationship has—to our knowledge—not previously been recognized.

For a potential explanation of this phenomenon one should consider that primary YCs and oil concentration can be altered by the source–sink relationship at different developmental stages. While resources at pre-flowering influence the number of siliques and the number of potential seeds per silique, seed size develops after flowering and fatty acid biosynthesis also takes place ~15–35 d after flowering, and thus both are dependent on assimilate availability at this stage (Tzen *et al.*, 1993). TSW is known to be able to compensate in cases where other primary YCs are underdeveloped as a consequence of earlier assimilate constraints (Labra *et al.*, 2017). Thus, after the flowering period, a trade-off in sink capacity can occur. Since oil is a very light but energy-dense substance, genotypes high in seed oil concentration can consequently devote less excess energy to the remaining seed components than genotypes which have a comparable flux of assimilates but less energy investment into seed oil. Furthermore, it is known that the end of flowering can directly influence both oil concentration and TSW (Hua *et al.*, 2012). Indeed, we observed a negative correlation between the end of flowering and TSW (*r*= –0.43 at low N and *r*= –0.45 at high N). In contrast, a corresponding relationship was not found between the end of flowering and seed oil concentration.

### Consideration of primary yield components and leaf N concentration in future breeding programs

For estimation of breeding progress, heritability is an important parameter to calculate the response to selection. In our study, we found that the number of siliques on the MR (*h*^2^=0.81) and TSW (*h*^2^=0.93) had a higher heritability than seed yield on the MR (*h*^2^=0.71) and SBs (*h*^2^=0.42), suggesting that selection on single YCs might lead to a higher selection response. This is in agreement with findings from genetic studies where heritabilites for single traits (number of siliques per plant, *h*^2^=0.69; number of seeds per silique, *h*^2^=0.87; and TSW, *h*^2^=0.93) were higher than the heritability of seed yield (*h*^2^=0.64), and quantitative trait loci (QTLs) for single traits are more stable across environments than QTLs for seed yield (Cai *et al.*, 2016). Although it has been suggested that low heritability of silique number per plant represses selection response for this trait (Richards and Thurling 1979), our data demonstrate that hybrid varieties in fact have a considerable advantage in this trait. In contrast to the number of siliques, the number of seeds per silique or likewise the silique length have been proposed as parameters for indirect selection to improve yield (Chay and Thurling, 1989). Correspondingly, our results clearly demonstrate that, irrespective of the variety type (OP or hybrid), the number of seeds per silique has increased significantly throughout the last two decades of breeding history (Fig. 5), even though the heritability for this trait is low (Table 4). The fact that seed yield measured by threshing of whole plots leads to a higher heritability than those of individual YCs is most probably influenced by sample size in terms of sampled plants. Determination of seed yield on a plot scale (including hundreds of plants) is more precise than estimation based on three single plants, even when the chosen plants are representative for the plot. On the other hand, selection based on consideration of individual YCs is probably hampered by a lack of suitable methods to phenotype complex plant architecture traits during high throughput. Overcoming these hurdles is of utmost importance to better understand the role of plant architecture in yield performance and better incorporate available genetic variation.

Our results also demonstrated an association of leaf N status at flowering with seed oil concentration at maturity, especially under low N fertilization. Since N is an essential nutrient with an overwhelming importance in the photosynthesis apparatus, one can argue that higher leaf N leads to a higher photosynthetic activity, which in turn enhances the quantity of assimilates allocated to the final sink, contributing to oil biosynthesis. In one recent study it was found that an increased storage N pool in the plant biomass results in leaf expansion and a higher photosynthetic capacity (Liu *et al.*, 2018). Together with other studies describing the relationship between photosynthetic capacity and leaf N content (Rotundo and Cipriotti, 2017), these observations support our hypothesis of an increasing assimilate source at a high N status. Leaf N status is a widely used and easy to measure indicator of plant N status. For both the leaf N concentration at flowering and the seed oil concentration, we found highly significant genotype effects. This is of considerable interest for future studies. Since the C and N concentrations of leaves show the highest heritability of the vegetative plant traits, one would expect a certain scope for further breeding gains. Also, further studies are required to check the within-species genetic variation in light-saturated photosynthesis in relationship to leaf N per unit leaf area.

### Conclusion

Higher plant biomass until flowering and increase of number of seeds per plant were identified as the major contributors for higher seed yield, and thus enhanced NUE in oilseed rape, one of the world’s most important non-N-fixing, docotyledonous crops. Higher Nup associated with higher numbers of siliques per plant can be achieved by breeding hybrid varieties. Furthermore, productivity of individual siliques has been achieved by enhancement of the number of seeds per silique. We argue that additional consideration of TSW in selection decisions might help to increase seed yield further, in particular under low N supply. Given the broad genetic variation available for TSW, along with the fact that it has the highest heritability among all yield components in our and other studies (Radoev *et al.*, 2008), including TSW in selection decisions would probably increase the response to selection and/or might help avoid negative co-selection. In a recent study it was discussed that an increase in TSW without negative effects on oil concentration is feasible (Labra *et al.*, 2017). Future studies should clarify whether considering TSW in selection decisions can also increase the productivity of individual siliques. In this regard, increased understanding of compensatory effects between primary YCs is essential to better understand and change determinations for architecture. For elucidation of plant N response across the entire vegetation, destructive measurements are not appropriate. Instead non-destructive high-throughput techniques, for example by unmanned aerial vehicles, can be a feasible option. Those data, coupled with elucidation of genome diversity (Voss-Fels and Snowdon, 2016), coupled with identification and application of haplotypes (Qian *et al.*, 2017), facilitate a better understanding of genetic determinants of leaf N concentration and plant architectural traits under low N input in oilseed rape. Coupled with algorithms that enable genome-based predictions of complex trait expression (Würschum *et al.*, 2014; Jan *et al.*, 2016; Werner *et al.*, 2018) in conjunction with growth models (Technow *et al.*, 2015), there is considerable scope for further breeding progress towards higher yielding and thus more efficient dicotyledonous crop varieties.

## Supplementary data

Supplementary data are available at *JXB* online.

Fig. S1. Average adjusted means of thousand seed weight (TSW) for individual variety groups determined over six environments at (A) high and (B) low nitrogen fertilization.

Fig. S2. Average adjusted means for density of siliques on side branches for individual variety groups determined over six environments at (A) high and (B) low nitrogen fertilization.

Fig. S3. Average adjusted means of number of siliques per branche for side branches at (A) high and (B) low nitrogen fertilization.

Fig. S4. Inter-trait correlations among all investigated traits at (A) low and (B) high nitrogen fertilization.

Table S1. Groups of investigated varieties according to year of registration and type of variety.

Table S2. Environment specific soil conditions (Stahl *et al.*, 2017).

Table S3. List of investigated traits, their abbreviation, and overview of environments in which the data were collected.

Table S4. Number of investigated varieties and total number of analyzed plants for yield components after elimination of outliers (±2.5 SD from the overall mean).

Supplementary Figures S1-S4

Supplementary Tables S1-S4

## Abbreviations:

doy: day of the year
MR: main raceme
Nup: nitrogen uptake
NutE: nitrogen utilization efficiency
NUE: nitrogen use efficiency
OP varieties: open pollinated varieties
SB: side branch
TSW: thousand seed weight
YC: yield component.
