# Supplementary material for: Effect of breeding on nitrogen use efficiency-associated traits in oilseed rape
Source: J Exp Bot. 2019 Feb 9;70(6):1969–86. doi: 10.1093/jxb/erz044 (PMC6436158; doi:10.1093/jxb/erz044)
Supplement: Supplementary Figures S1-S4 [file erz044_suppl_supplementary_figures_s1-s4.pdf]

Effect of breeding on nitrogen use efficiency associated traits in oilseed rape

Andreas Stahl[1*], Paul Vollrath[1], Birgit Samans[1], Matthias Frisch[2], Benjamin Wittkop[1] and Rod J. Snowdon[1]

[1] Department of Plant Breeding, IFZ Research Centre for Biosystems, Land Use and Nutrition, Justus Liebig University, Heinrich Buff-Ring 26-32, 35392 Giessen, Germany

[2] Department of Biometry and Population Genetics, IFZ Research Centre for Biosystems, Land Use and Nutrition, Justus Liebig University, Heinrich Buff-Ring 26-32, 35392 Giessen, Germany

Supplementary Figures

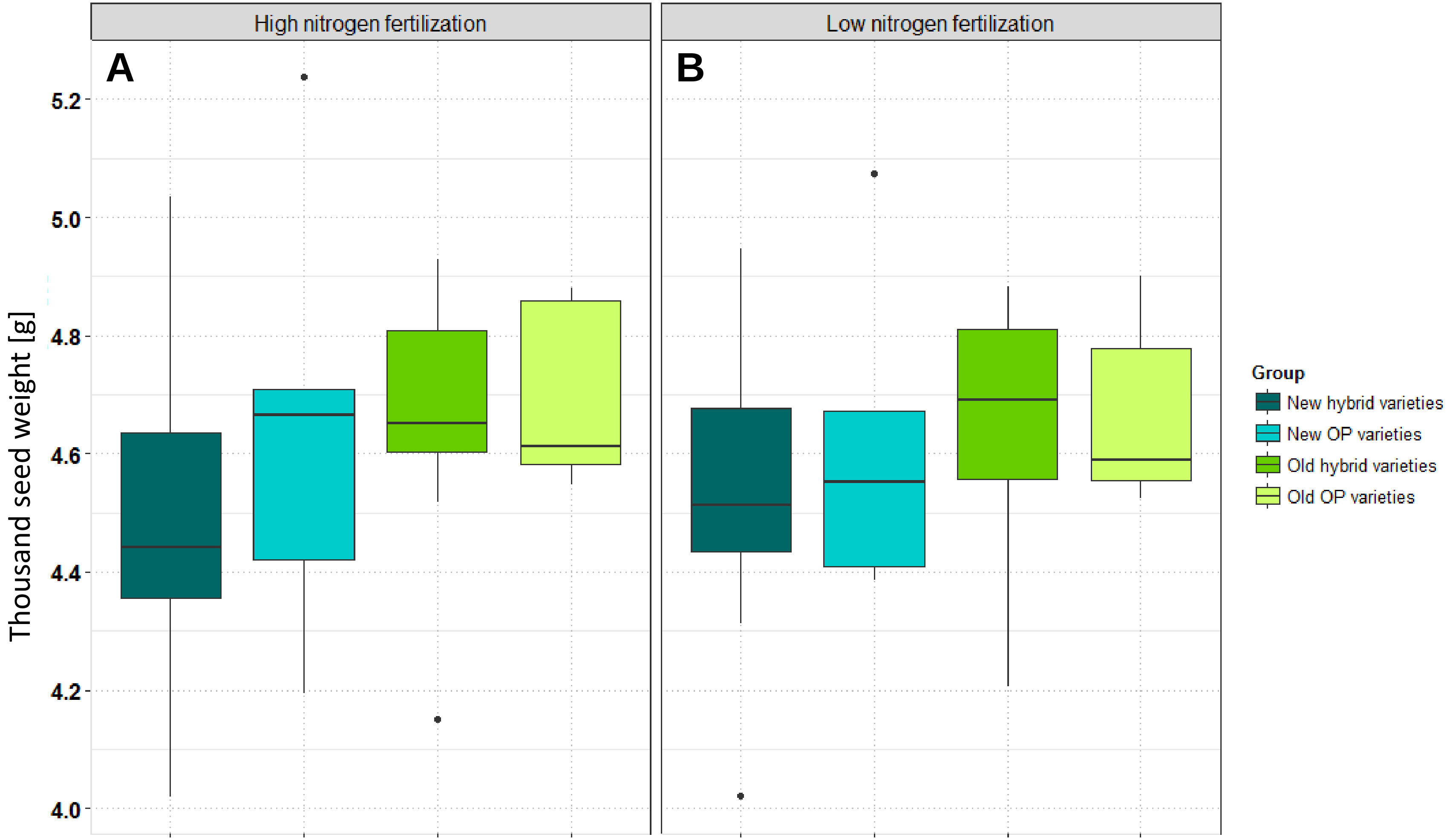

Supplementary Figure 1: Average adjusted means of thousand seed weight (TSW) for individual variety groups determined over six environments at (A) high and (B) low nitrogen fertilization.

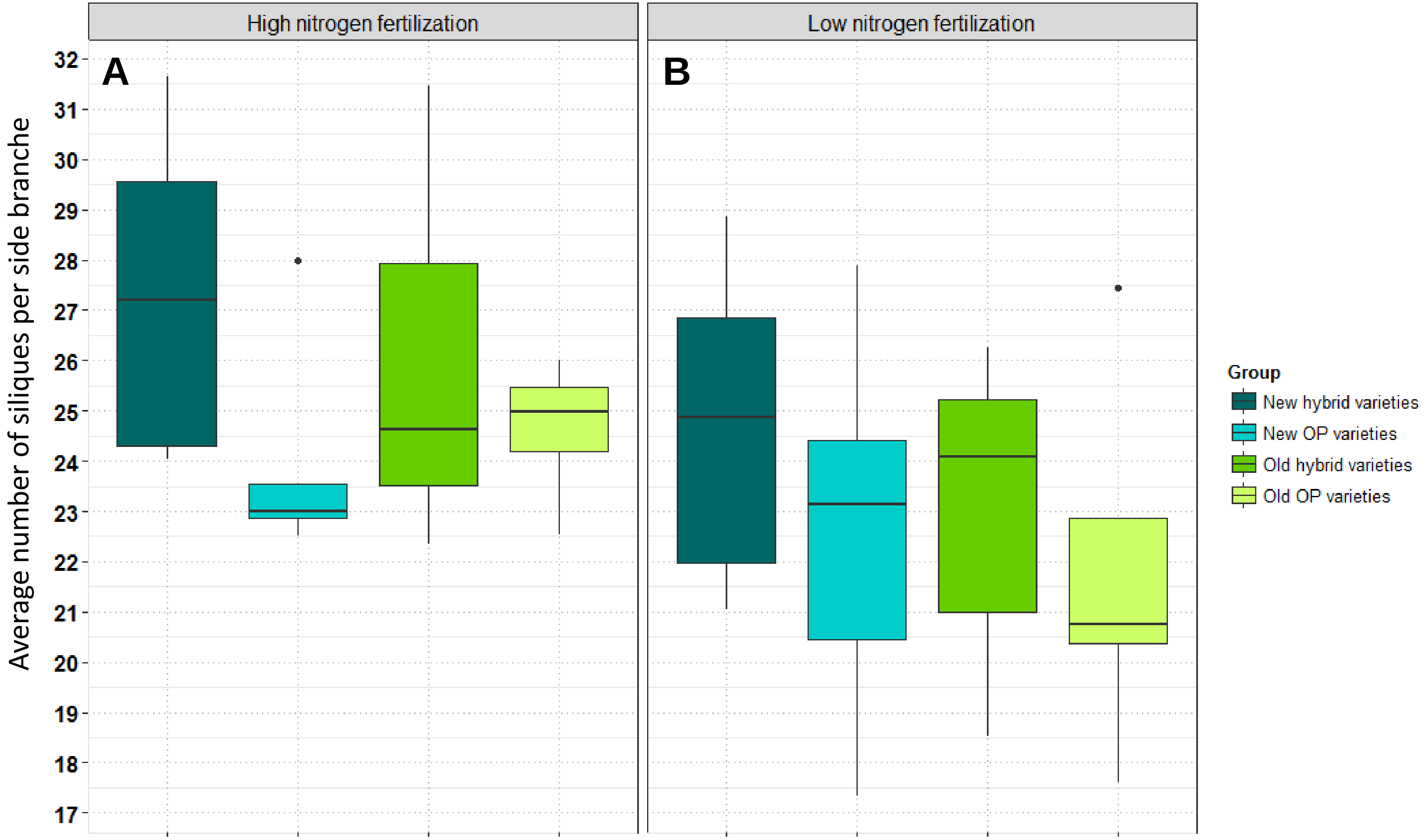

Supplementary Figure 2: Average adjusted means for density of siliques on side branches for individual variety groups determined over six environments at (A) high and (B) low nitrogen fertilization, respectively.

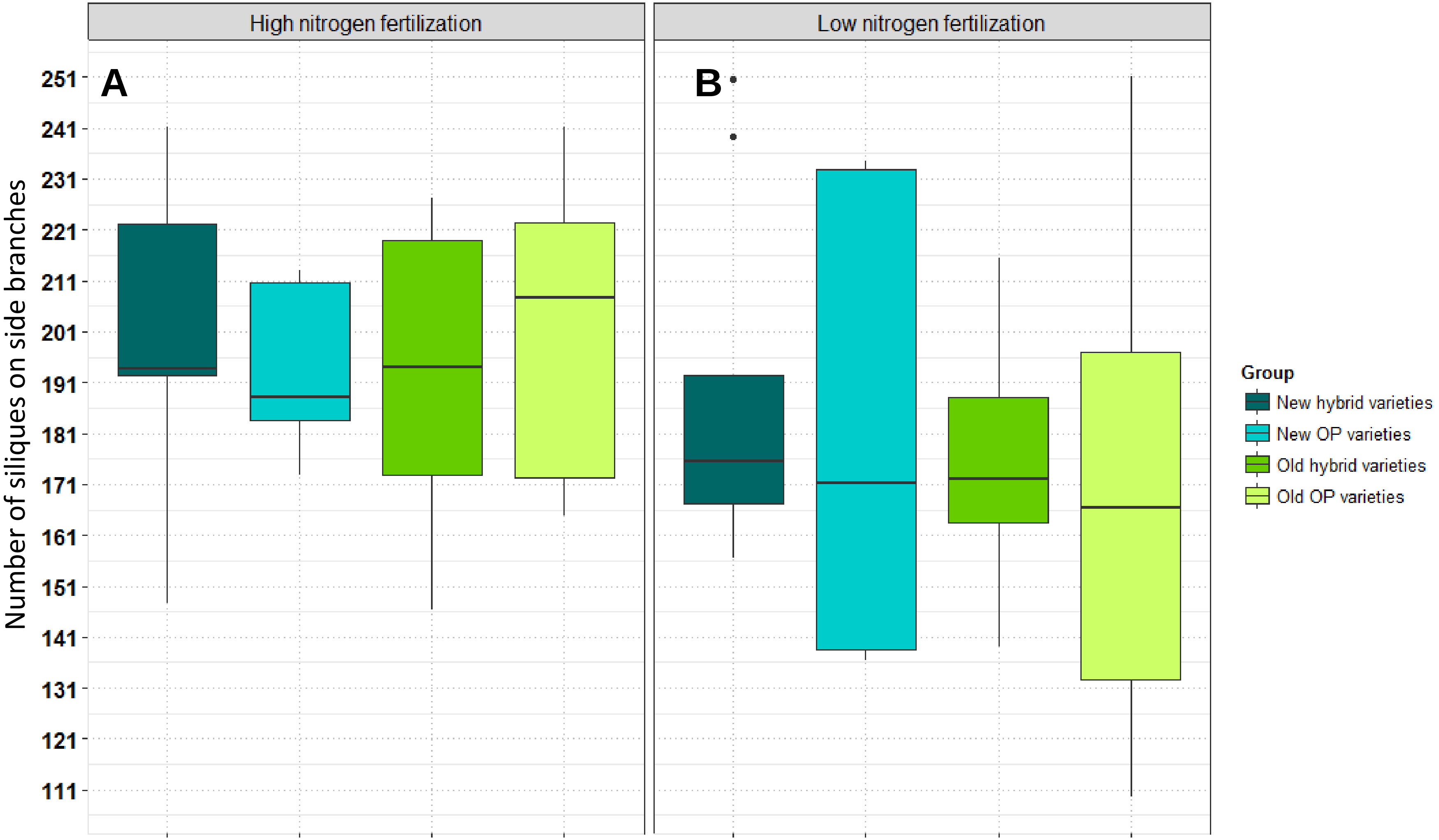

Supplementary Figure 3: Average adjusted means of number of siliques per branche for side branches at (A) high and (B) low nitrogen fertilization, respectively.

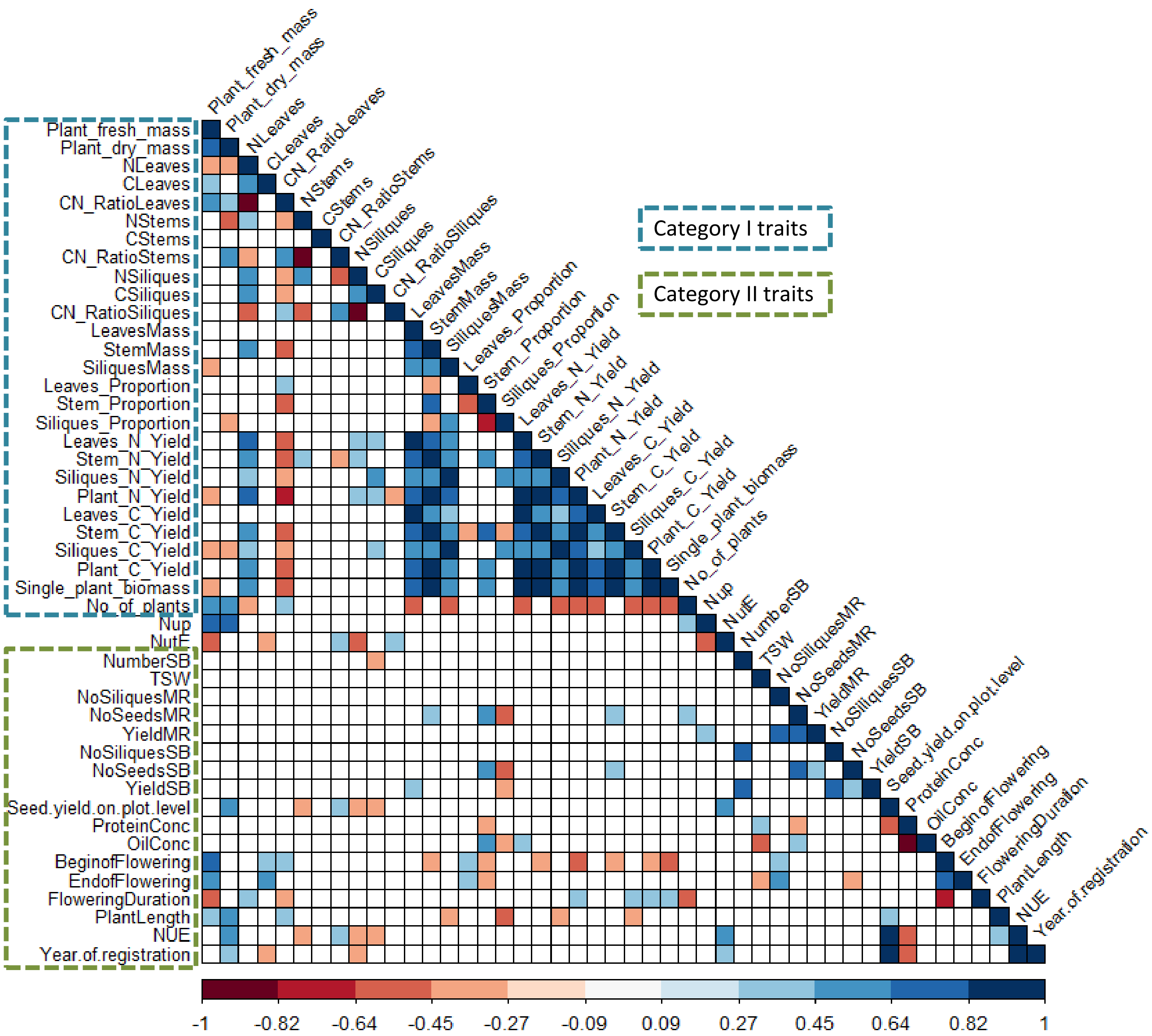

Supplementary Figure 4a: Inter-trait correlations among all investigated traits at low nitrogen fertilization. Direction and strength of Pearson's coefficient of correlation is depicted by the color code. Non-significant correlations are shown as white boxes.

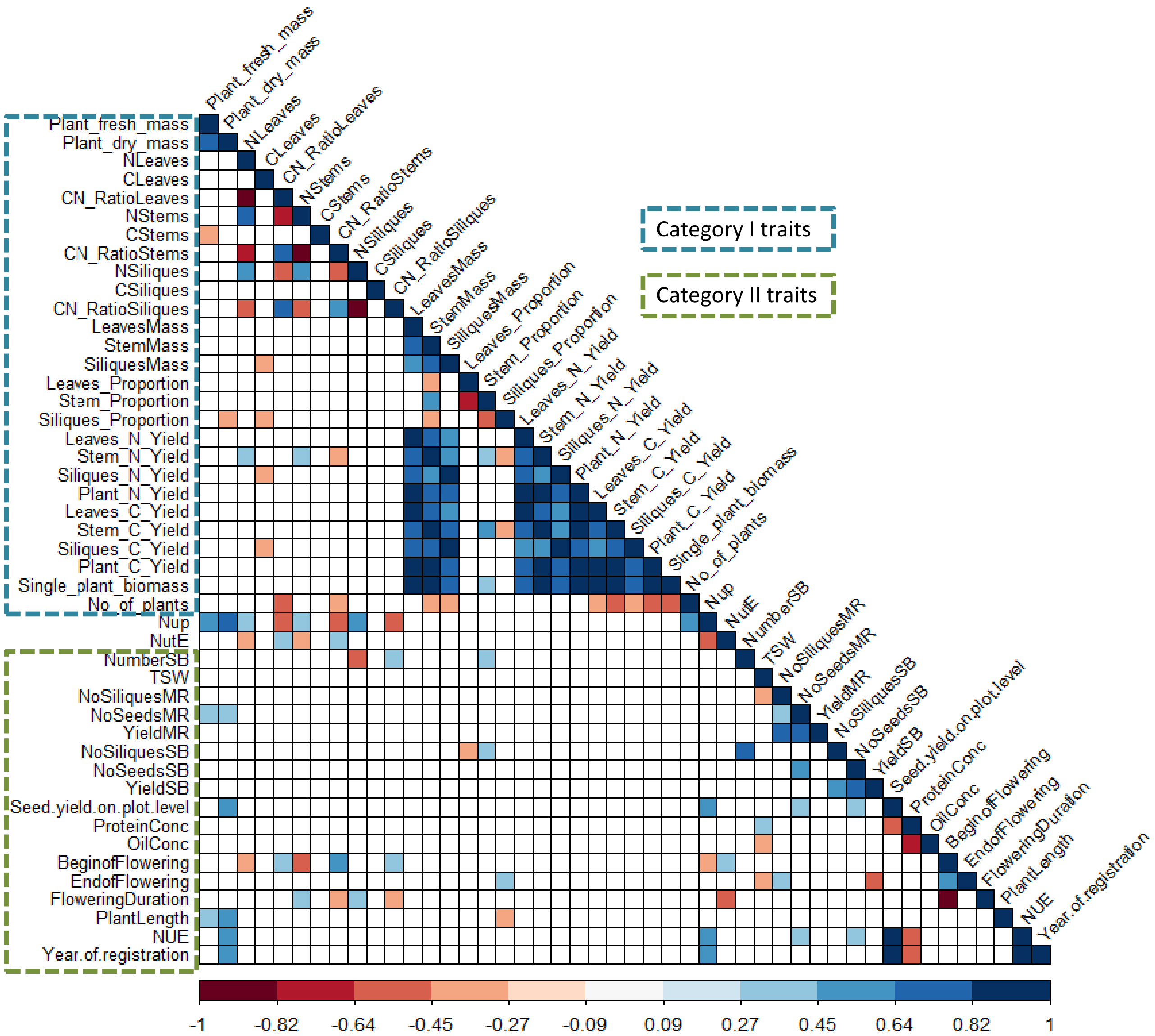

Supplementary Figure 4b: Inter-trait correlations among all investigated traits at high nitrogen fertilization. Direction and strength of Pearson's coefficient of correlation is depicted by the color code. Non-significant correlations are shown as white boxes.
