# Supplementary material for: Effect of breeding on nitrogen use efficiency-associated traits in oilseed rape
Source: J Exp Bot. 2019 Feb 9;70(6):1969–86. doi: 10.1093/jxb/erz044 (PMC6436158; doi:10.1093/jxb/erz044)
Supplement: Supplementary Tables S1-S4 [file erz044_suppl_supplementary_tables_s1-s4.pdf]

Stahl et al.,

Effect of breeding on nitrogen use efficiency associated traits in oilseed rape

Supplementary Tables

Supplementary Table 1: Groups of investigated varieties according to year of registration and type of variety

**New hybrid varieties**	**Year of registration**
Thure (SDH)	2014
Marathon	2013
Mercedes	2013
Avatar	2011
DK Exstorm	2011
Inspiration	2011
Genie	2011
Mascara	2011
Troy (SDH)	2011
**Old hybrid varieties**	
Artoga	2010
Sherpa	2010
Compass	2009
NK Linus	2009
Visby	2007
Exocet	2005
Taurus	2004
Baldur	2002
Elektra	2002
Ryder	2000
Artus	1997
**New OP varieties**	
Patron	2012
Trinity	2012
Adriana	2007
Lorenz	2005
Oase	2004
**Old OP varieties**	
Pacific	2003
Californium	2002
Aviso	2000
Express	1993
Lirajet	1989

SDH = Semi dwarf hybrid

Supplementary Table 2: Environment specific soil conditions (Stahl et al., 2017)

**Environment**	**P_2O_5 [mg/100g]**	**K_2O [mg/100g]**	**Soil pH**	**Soil Type according to FAO, yield index units**	**N_{min} at beginn of vegetation 0-60cm [kg N/ha]**	**Plot size (harvested area) [m^2]**
ASE15	14.8	15	8.1	silty loam	47	12
ASE16	7.5	10	NA	loamy sand, aprox. 45	29	12
MOS15	10.9	13	7.1	loessic loam	21	10.2
MOS16	12.0	18	NA	loessic loam	26	10.2
RHH15	10.4	6	7.6	loam, 80	25	10.5
RHH16	10.6	7	7	loam, 85	13	10.5

Supplmentary Table 3: List of investigated traits, their abbreviation and overview of environments in which the data were collected.

category	Abbreviation	Trait	ASE2015	ASE2016	RHH2015	RHH2016	MOS2015	MOS2016
I	PlantFreshMass	Plant fresh mass on plot level						
I	PlantDryMass	Plant dry mass on plot level						
I	N conc Leaves	N concentration in leaves					*	
I	C conc Leaves	C concentration in leaves					*	
I	CN RatioLeaves	C/N ratio in leaves					*	
I	N conc Stems	N concentration in stems					*	
I	C conc Stems	C concentration in stems					*	
I	CN RatioStems	C/N ratio in stems					*	
I	N conc Siliques	N concentration in siliques					*	
I	C conc Siliques	C concentration in siliques					*	
I	CN RatioSiliques	C/N ratio in siliques					*	
I	LeavesMass	Leaves mass at flowering on plant level					*	
I	StemMass	Stem mass at flowering on plant level					*	
I	SiliquesMass	Siliques mass at flowering on plant level					*	
I	LeavesProportion	Proportion of leaves on entire plant dry mass					*	
I	StemProportion	Proportion of stems on entire plant dry mass					*	
I	SiliquesProportion	Proportion of siliques on entire plant dry mass					*	
I	LeavesNYield	N mass in leaves at flowering					*	
I	StemNYield	N mass in stems at flowering					*	
I	SiliquesNYield	N mass in siliques at flowering					*	
I	PlantNYield	N mass in entire plant at flowering					*	
I	LeavesCYield	Cmass in leaves at flowering					*	
I	StemCYield	C mass in stems at flowering					*	
I	SiliquesCYield	C mass in siliques at flowering					*	
I	PlantCYield	C mass in entire plant at flowering					*	
I	SinglePlantBiomass	Dry weight of single plant					*	
I	NoOfPlants	Number of plants per square meter					*	
II	NoSB	Number of side branches						
II	TSW	Thousand seed weight						
II	NoSiliquesMR	Number of siliques on main raceme						
II	NoSeedsMR	Number of siliques on main raceme						
II	YieldMR	Yield on main raceme						
II	NoSiliquesSB	Number of siliques on side branches						
II	NoSeedsSB	Number of siliques on side branches						
II	YieldSB	Yield on side branches						
II	Seed Yield	Seed Yield (SY)						
II	ProteinConc	Seed protein concentration at 91% dry mass						
II	Oilconc	Seed oil concentration at 91% dry mass						
II	BeginofFl	Begin of flowering						
II	EndofFl	End of flowering						
II	FlowDuration	Duration of flowering						
II	Pllength	Plant length						
II	NUE	Nitrogen use efficiency						
I	Nup	Nitrogen uptake efficiency						
I	NutE	Nitrogen utilization efficiency						

Grey shaded areas indicate those environments were data were collected.

* Data of high nitrogen treatment were collected only in one replication in this particular environment. Plant partitioning into ground stem, leaf and silique samples and subsequent C/N analysis was conducted for low N treatment at both locations in both years (n=12) and for high treatment at RHH2015 in three replications and additionally in MOS2015 in one further replication (n=4).

Supplementary Table 4: Number of investigated varieties (white) and total number of analyzed plants (grey) for yield components after elimination of outliers (+- 2.5 SD from the overall mean). MR: Main raceme, SB: side branches

Year	Loccation	NFL	NoSB	TSW	NoSiliques MR	Yield MR	NoSeeds MR	NoSiliques SB	Yield SB	NoSeed SB
2015	ASE	1	30	30	30	30	30	30	30	30
2015	ASE	2	30	30	30	30	30	30	30	30
2015	MOS	1	30	30	30	27	30	29	27	30
2015	MOS	2	30	30	30	27	28	29	27	28
2015	RHH	1	30	30	30	30	30	30	30	30
2015	RHH	2	30	30	30	30	30	30	30	30
2016	ASE	1	29	29	29	29	29	29	29	29
2016	ASE	2	29	29	29	29	29	29	29	29
2016	MOS	1	29	29	29	29	29	29	29	29
2016	MOS	2	29	29	29	29	29	29	29	29
2016	RHH	1	25	29	26	26	26	26	26	26
2016	RHH	2	29	29	29	29	29	29	28	29
Total number of plants			1041	1059	1036	1031	1029	1022	1018	1021
